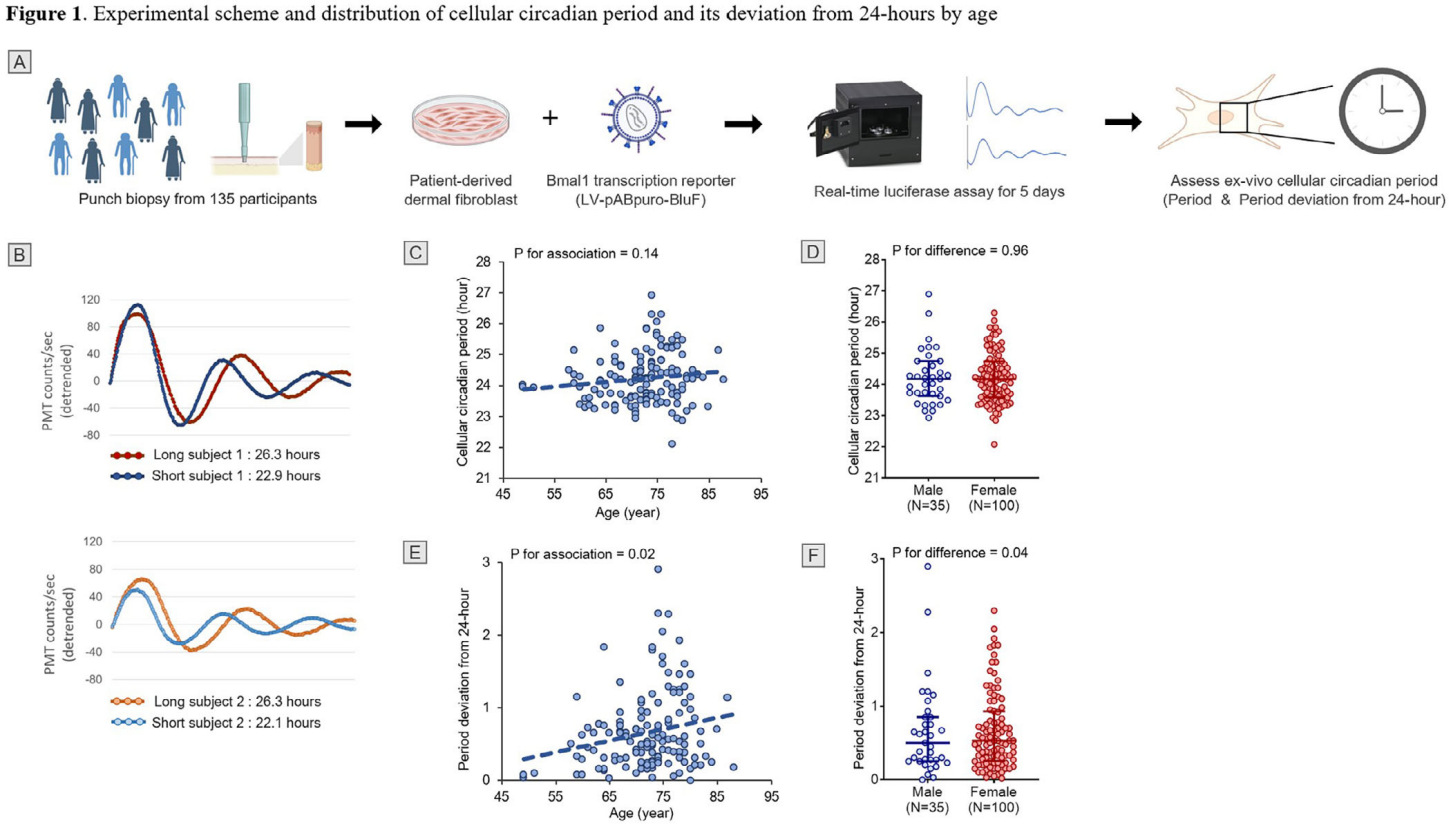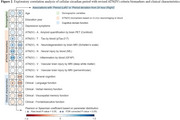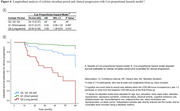# Associations of ex‐vivo cellular circadian period with aging, tau, neurodegeneration, inflammation and clinical progression in older adults with cognitive impairment: Findings from BICWALZS cohort

**DOI:** 10.1002/alz70855_100114

**Published:** 2025-12-23

**Authors:** Hyun Woong Roh, Sunwoo Yoon, Homin Song, You Jin Nam, Sunhwa Hong, Eun Young Kim, Sang Joon Son, Chang Hyung Hong

**Affiliations:** ^1^ Ajou University School of Medicine, Suwon, Gyeonggido, Korea, Republic of (South)

## Abstract

**Background:**

Alterations in circadian rhythms are widely observed in Alzheimer's disease (AD) and other neurodegenerative disorders. However, the significance of ex‐vivo cellular circadian periods and their deviation from the 24‐hour cycle, measured in patient‐derived fibroblasts, remains largely unexplored.

**Method:**

We analyzed 135 older adults with cognitive complaints from the BICWALZS cohort. Cellular circadian periods and their deviation from 24 hours were measured in patient‐derived dermal fibroblasts using a lentiviral vector reporting Bmal1 transcription via real‐time luciferase assays. Biomarkers reflecting revised ATN(IV) criteria for AD, including plasma biomarkers and neuroimaging, were assessed. Exploratory correlation analyses and generalized linear models adjusted for covariates identified associations. Voxel‐based morphometry explored regional grey matter density, and survival analysis evaluated longitudinal clinical progression.

**Result:**

The cellular circadian period was significantly associated with tau proteinopathy (plasma pTau‐217, *p* = 0.012, small effect size), neural injury and degeneration (plasma NfL, *p* <0.001, large effect size; MRI Schelten's scale, *p* = 0.033, small effect size), and inflammation (plasma GFAP, *p* <0.001, medium effect size). Longer periods correlated with reduced grey matter density in AD‐relevant regions, including the left and right amygdala (*p* = 0.027 and *p* = 0.040, respectively) and the left parahippocampal gyrus (*p* = 0.042). Deviation from 24 hours was strongly associated with age (*p* = 0.02, FDR‐p=0.06), neurodegeneration (MRI Schelten's scale, *p* = 0.004), cognitive decline (MMSE, *p* = 0.016), and memory and language impairments (all *p* <0.05). Voxel‐based morphometry revealed broader associations for period deviation with grey matter density in aging‐ and neurodegeneration‐related regions, including the hippocampus and superior/middle temporal poles. Survival analysis showed that longer cellular circadian period were associated with faster clinical progression (HR=3.59, *p* = 0.01).

**Conclusion:**

Cellular circadian metrics, particularly period deviation, reflect aging‐related neurodegenerative changes and cognitive impairments, while longer periods are linked to AD‐specific pathology and faster clinical progression. These findings highlight their potential as biomarkers for AD and aging‐related neurodegeneration, warranting further investigation.